# Examining the link between coach-athlete relationship and athlete burnout among college soccer players: the mediating role of training satisfaction

**DOI:** 10.3389/fpsyg.2024.1409609

**Published:** 2024-08-06

**Authors:** Liu Jiahao, Li Jing

**Affiliations:** ^1^School of Sports Training, Chengdu Sport University, Chengdu, Sichuan, China; ^2^School of Physical Education, Sichuan University, Chengdu, Sichuan, China

**Keywords:** athlete burnout, coach-athlete relationship, training satisfaction, college athletes, soccer players

## Abstract

**Purpose:**

Athlete burnout is an adverse factor that usually decreases athletes' sports performance and reduces their passion for athletic career development. The present study examined the association of coach-athlete relationship, training satisfaction, and athlete burnout, and then explored the training satisfaction as a mediating role of the effect of coach-athlete relationship on athlete burnout among college soccer players.

**Methods:**

Soccer players from seven Chinese higher education institutions were recruited for the study. The participants were selected using stratified random sampling and included 218 participants. The Coach-Athlete Relationship Scale, Training Satisfaction Scale, and Athlete Burnout Questionnaire were used to collect data from the participants. Pearson's product-moment correlation analysis, linear regression analysis, and bootstrap method were conducted to analyze the collected data set.

**Results:**

The results showed that (1) there was a significant correlation between coach-athlete relationship, training satisfaction, and athlete burnout. (2) coach-athlete relationship and training satisfaction significantly negatively predicted athlete burnout (β = −0.39, *p* < 0.001; β = **-** 0.29, *p* < 0.001). (3) training satisfaction had a significantly mediating effect on the relationship between coach-athlete relationship and athlete burnout (β = −0.15, *p* < 0.001, ES = 0.28).

**Conclusion:**

Coach-athlete relationship can not only directly negative impact athlete burnout but can also negatively influence athlete burnout through the mediating effect of training satisfaction. Therefore, we suggest the following to alleviate athlete burnout: on one hand, coaches can provide more communication opportunities for athletes to improve the coach-athlete relationship. On the other hand, teams can enhance training satisfaction by building team culture.

## Introduction

Athlete burnout refers to the phenomenon of decreased psychological functioning in athletes when facing various pressures (Zhang et al., 2006). If athletes have been suffering burnout for a long time, the athlete burnout can have adverse effects on their daily lives or even their entire professional careers (Maslach, 1984). The concept of burnout was initially introduced by Maslach (1984), with his research primarily focusing on professions in the service industry, including volunteers, teachers, and nurses. These occupations involve extensive interactions with individuals and require a significant emotional commitment toward others. The continuous engagement within such environments often triggers adverse emotional responses, such as feelings of sadness and diminished self-assurance among workers. After the 1980s, burnout emerged as a prominent research subject for psychologists and sociologists. Gradually, the term “burnout” is becoming increasingly widely used in the field of sports. Athlete burnout was introduced by Raedeke (1997), which relates to the adverse mental state athletes experience that results from prolonged, high-intensity, repetitive training. Such demanding training exposes athletes to varying levels of psychological strain, ultimately leading to the development of negative psychological conditions. There are three sub-scales in athlete burnout: emotion/physical exhaustion, reduced athletic accomplishment, and sport devaluation (Madigan et al., 2022). Scholars have been exhibiting a growing interest in studying burnout among athletes in recent years (Isoard-Gautheur et al., 2016). Research suggests that athlete burnout might result in dropout behavior, and impede the wellbeing and motivation of athletes (Martinent et al., 2014; Graa et al., 2021; Nicholls et al., 2022). China's soccer training is deeply rooted in the country's culture of discipline and hard work. It typically emphasizes strict discipline between coaches and players and involves high-intensity training, leading to soccer players enduring burnout. Moreover, in the competitive landscape of college soccer competitions in China, young collegiate athletes, whose mental development is still ongoing, are more likely prone to experiencing burnout (Zhang, 2010; Guo et al., 2021). Nevertheless, athlete burnout is often disregarded within the student-athlete community, despite it becoming increasingly common in college soccer in China. High levels of burnout can indeed impact the mental health of collegiate athletes, while also posing a threat to the healthy development of college soccer in China (Zhao et al., 2022). Therefore, it is essential to explore the mechanisms of athlete burnout within collegiate soccer players in China. By expanding our theoretical understanding of the causes of athlete burnout, we can prevent or alleviate athlete burnout and develop practical solutions to mitigate its impact.

Regarding the formation of athlete burnout, Smith (1986) believed that when athletes assess that the demands of a situation either do not align with their available resources or surpass their ability to cope with those demands, stress is activated. This persistent imbalance between perceived demands and available resources results in the emergence of burnout. With the ongoing advancement of research, researchers have increasingly recognized that the factors contributing to athlete burnout are negative training experiences and stress. Raedeke and Smith (2001) believed that prolonged monotonous training can lead to emotional and physical exhaustion in athletes, while excessively difficult goals can decrease athletes' sense of achievement. Additionally, high competitive pressure and low social support both significantly increase the level of athlete burnout (Lin et al., 2021; Martinez-Alvarado et al., 2021; Shang and Yang, 2021). The perspective that athlete burnout results from a combination of external and internal factors is gaining more attention (Zhang et al., 2006; Chen and Zhou, 2008). On the one hand, researchers found that athletes are prone to experiencing athlete burnout as a result of external factors, such as social organizational format and coach-athlete relationship dynamics and over training (Zhang, 2010; DeFreese and Smith, 2014; Guo et al., 2021). On the other hand, some scholars argue that internal factors within individuals are significant contributors to athlete burnout. Cresswell and Eklund (2005) believed that long-term dissatisfaction of athletes' three fundamental psychological needs, namely competence, autonomy, and relatedness, in the sporting context is a crucial factor contributing to athlete burnout. Besides, previous studies have found that perceived reward has a positive and significant correlation with athlete burnout, while self-perceived ability, personality traits, and perfectionism are negatively correlated with athlete burnout (Maslach, 1984; Cresswell and Eklund, 2005; Li and Zhang, 2013).

Some models explain the causes of athlete burnout by considering a combination of internal and external factors. According to training stress syndrome model, negative adaptations to training are inevitable for individuals (Silva, 1990). Factors such as excessive pressure and negative training experience can result in negative adaptations. When negative adaptations reach a point where the body cannot effectively cope, it can lead to athlete burnout and, in extreme cases, even prompt individuals to withdraw from training (Silva, 1990). Meanwhile, the cognitive-affective model suggests that burnout develops parallel to stress. When individuals perceive that they cannot meet environmental needs such as demands of high training requirements, they may experience stress, burnout, and other negative psychological responses (Smith, 1986). Therefore, how to cope with stress and enhance training quality are key factors influencing athlete burnout. Throughout the athlete's developmental journey, coaches are the most crucial sources of social support for athletes, play a vital role in helping athletes effectively cope with pressure and maintain their physical and mental wellbeing, especially in the face of intense competition and high-pressure training (Du et al., 2021). Research has found that a positive coach-athlete relationship is the main factor for athletes to receive effective support (Miao et al., 2022), while the support from coaches can significantly improve athletes' satisfaction with training and competition (Guo and Yang, 2021). Based on this, the coach-athlete relationship is a key underlying factor for reducing and effectively alleviating negative training adaptations, thereby reducing athlete burnout. Previous research has primarily focused on the relationship between pressure and athlete burnout. However, there has been limited exploration that elaborates on the specific impact of coaches on athletes' psychological fatigue from a negative adaptation perspective (Qiu and Zhang, 2019; Li et al., 2024). Moreover, based on the training stress syndrome model, accurately understanding athletes' evaluations of training quality is equally crucial for preventing athlete burnout. In light of this, both the need for meaningful relationships between coaches and athletes and the quality of training experiences emerge as pivotal factors impacting athlete burnout. Hence, it is essential to conduct further research and exploration in this area.

### Coach-athlete relationship and athlete burnout

Positive interpersonal relationships can enhance overall satisfaction among athletes (Chelladurai, 1984). Coach-athlete relationship holds paramount significance in an athlete's professional journey, as it directly influences their satisfaction with sports and their performance (Jowett and Lavallee, 2007; Jowett and Arthur, 2019; Steven et al., 2019; Li et al., 2021). The relationship is an interaction between coaches and athletes in terms of their feelings, perceptions, and behaviors (Jowett and Lavallee, 2007; Jowett, 2009). It is serves as the primary link in training, rooted in the common objectives of athletic competition. Some scholars believed that within the Chinese sports system, the “coach-athlete relationship” is established on a foundation of ethics and hierarchy, representing a social relationship (Xie, 2015). A positive coach-athlete relationship can offer timely assistance to athletes when needed and assist athletes in coping with stressful environments, thereby enhancing athletes' subjective perception of support (Miao et al., 2022). Studies have demonstrated that the coach-athlete relationship has a direct impact on athlete burnout (Zhang et al., 2010; Xie, 2013; Isoard-Gautheur et al., 2016). This maybe because a positive coach-athlete relationship can provide athletes with more opportunities for interaction with their coaches. Coaches can offer support to athletes through these interactions, thus positively impacting athletes' physical and psychological wellbeing. When it comes to studies concerning the coach-athlete relationship and burnout, the majority have predominantly centered on the coach's viewpoint, while research conducted from the athlete's perspective remains limited (Barcza-Renner et al., 2016). Therefore, this study aimed to assess the coach-athlete relationship from the perspective of athletes, providing coaches with insights into athletes' subjective experiences in daily interactions. Additionally, it seeks to offer suggestions for improving interaction patterns.

### Training satisfaction and athlete burnout

Athletes' satisfaction with themselves is often a predictor of their mental wellbeing (Xie and Yao, 2010). Previous research has found a negative correlation between satisfaction and burnout (Bu et al., 2020), and training satisfaction is associated with competitive performance and athlete burnout (Zhan et al., 2015). Training satisfaction pertains to an athlete's personal perception and assessment of their experiences during training and competitions (Zhan et al., 2015). Training satisfaction, as subjective cognitive assessments for athletes, play a crucial role in influencing emotional states and self-efficacy. Through positive experiences and high levels of satisfaction, athletes can regulate their emotions, reduce the occurrence of negative emotions, and maintain a positive attitude when facing challenges (Bu et al., 2020). This positive mindset helps alleviate burnout, enhance athletes' psychological resilience, and consequently improve their performance levels in both training and competition. Training satisfaction can reflect the enjoyment of athletes during training and provide a genuine reflection of their training status. Therefore, this indicator serves as a crucial basis for coaches to control training pace.

### The moderating effect of training satisfaction

Based on the training stress syndrome, athletes' burnout is caused by excessive negative adaptation to training pressures (Silva, 1990). A positive coach-athlete relationship can provide athletes with interpersonal support, thereby alleviating negative adaptation. On the other hand, individuals with high levels of training satisfaction can mitigate the impact of stress. Based on this, training satisfaction not only directly affects psychological fatigue but also has the potential to enhance the coach-athlete relationship's ability to alleviate athlete burnout. Research has revealed that athletes' satisfaction with training is influenced by the coach-athlete relationship (Kardefelt-Winther, 2014; Elhai et al., 2020), where positive relationships can enhance athletes' satisfaction with training and competitions (Błachnio and Przepiorka, 2019; Sindermann et al., 2020). However, there has been limited exploration of whether the coach-athlete relationship can subsequently impact athlete burnout through the lens of training satisfaction. Furthermore, prior research has not adequately addressed the unique context of collegiate athletes within this athlete population (Wu and Zhao, 2002). To facilitate the advancement of competitive sports in China, we must direct more focus toward collegiate athletes (Gu, 2006).

### This research

The negative adaptation to pressure during training can reduce the training benefits for athletes, whereas interpersonal relationships and subjective perception of training quality, such as coach-athlete relationship and training satisfaction are key factors influencing the extent of negative adaptation among athletes. In addition, individual cognition varies, and subjective perceptions of training influence the occurrence of burnout. In Chinese college soccer league, at the college stage, high levels of athlete burnout have become increasingly prevalent. This trend poses significant challenges not only to the athletes' performance and wellbeing but also to the overall development of college soccer programs (Zhao et al., 2022). It is essential to investigate the factors contributing to this burnout to develop effective strategies for prevention and management. In summary, this research, based on the training stress syndrome and cognitive-affective model and approached from the standpoint of collegiate athletes, aimed to investigate the mechanisms of athlete burnout formation from both external and internal factors. Additionally, it seeks to explore the connections between the coach-athlete relationship, training satisfaction, and athlete burnout, while providing viable recommendations for mitigating burnout among collegiate athletes and making a modest contribution to the advancement of college soccer in China.

Although the relationship between athlete burnout, coach-athlete relationship, and training satisfaction has been thoroughly studied, there is still a lack of reports on the mediating role of training satisfaction between coach-athlete relationship and athlete burnout. This study aimed to investigate whether a positive coach-athlete relationship can mitigate negative training adaptation and, consequently, reduce the occurrence of burnout among college athletes. With these questions in mind, the following hypotheses are proposed:

(1) There is a significant negative correlation between coach-athlete relationship and athlete burnout.
(2) There is a significant positive correlation between coach-athlete relationship and training satisfaction.
(3) There is a significant negative correlation between training satisfaction and athlete burnout.
(4) Training satisfaction mediates the relationship between coach-athlete relationship and athlete burnout.

## Methods

### Participants

The participants of the present study were recruited from varsity soccer teams in seven universities. First, the players were classified according to their soccer teams, and then 321 students were randomly selected. A total of 321 questionnaires were distributed, and all questionnaires in the study were recovered. After analyzing and judgment from these responses, we excluded 103 cases for missing values or unreasonable values, finally, 218 cases were accepted in the analysis. The characteristics of participants in the study are described as follows (Table 1): there were 191 males (88%) and 27 females (13%). The age of participants ranged from 17 to 24 years old (Mean = 22.6, SD = 0.78). The average training experience (sports career) of participants was 3.4 years (SD= 1.23). In China, soccer proficiency is assessed using officially certified qualification levels issued by the General Administration of Sports. These levels are Master Sportsman, National Division 1, and National Division 2, representing players' soccer proficiency from high to low. In this study, there were 6 Master Sportsman participants (3%), 91 National Division 1 participants (42%), and 121 National Division 2 participants (55%).

**Table 1:** Demographic descriptive statistics of the respondents (*N* = 218).

**Variable**		**Number of cases**	**Percent**
Sex	Male	191	87.61%
	Female	27	12.39%
Grade	1	15	6.88%
	2	84	38.53%
	3	92	42.20%
	4	27	12.39%
Sports grade	Master sportsman	6	2.75%
	National level 1	91	41.74%
	National level 2	121	55.50%
**Age (years)**			
M ± (SD)		22.6	0.78
**Training experience**			
M ± (SD)		3.4	1.23

Continuous data number of cases and percent is mean ± (SD).

### Measures

We used a questionnaire that included demographic information of participants such as gender, age, and training experience, and three scales, The Coach-Athlete Relationship Scale, The Training Satisfaction Scale, and The Athlete Burnout Questionnaire.

#### The coach-athlete relationship scale

The scale was developed by Zhong and Wang (2007), including 13 items classified into three sub-scales: emotion (four items, e.g., “I respect my coach”), behavior (four items, e.g., “When I receive guidance from my coach, I feel cared for.”), and cognition (three items, e.g., “I am satisfied with the communication between my coach and me.”). These items were measured on a 7-point Likert scale, with 1 = “completely inconsistent” and 7 = “completely consistent”. It is scored in a positive direction. A high score indicates that the athlete perceives oneself a better relationship with his/her coach. Cronbach's coefficient of alpha (Cronbach's α) of this scale was 0.87 in this study, and Cronbach's α of its sub-scales were 0.76, 0.90, and 0.57 for emotion, behavior, and cognition, respectively. The KMO (Kaiser-Meyer-Olkin) of CAR is 0.84 with *p* < 0.001.

#### The training satisfaction scale

The scale has been developed by Zhang (2005), the scale includes six items as a single factor (e.g., “I am satisfied with my training and competitions.”). It was measured on a five-point Likert scale, with 1 = “strongly disagree”, and 5 = “strongly agree”. It is scored in a positive direction. A high score indicates that an athlete perceived better satisfaction with his/her training experience. Cronbach's α of this scale was 0.92 in this study. The KMO of TS is 0.92 with *p* < 0.001.

#### The athlete burnout questionnaire

The questionnaire was developed by Raedeke and Smith (2001) and translated into Chinese by Zhang and Mao (2004). The Chinese version comprises 15 items across three sub-scales: emotional/physical exhaustion (five items, e.g., “I feel so tired from my training that I have trouble finding energy to do other things.”), reduced athletic accomplishment (five items, e.g., “I don't feel confident about my sports ability.”), and sport devaluation (five items, e.g., “I don't care as much about my sports performance as I used to.”). The questionnaire was measured on a seven-points Likert scale, with 1 = “never”, and 7 = “always”. It is scored in a positive direction. A high score indicates that the athlete experienced more burnout in sports. Cronbach's α of this scale was 0.94 in this study, and the Cronbach's α of its sub-scales were 0.90, 0.88, and 0.89 for emotional/physical exhaustion, reduced athletic accomplishment, and sport devaluation, respectively. The KMO of ABQ is 0.94 with *p* < 0.001.

### Data preparation

A cross-sectional and voluntary sampling method was adopted in this study. The online survey including CAR, TS, and ABQ were made by the Questionnaire Star software package, and distributed to participants via a web link on the WeChat app with the help of their coaches. The participants completed the questionnaire with the guidance of their coaches and questionnaire instructions. The supervisors accompanied the coaches by real time video to ensure that the coaches left from the participants when they started writing the questionnaire. The whole process of filling out the questionnaire took ~25 min. Once finished, the questionnaire were automatically collected through the web link. The tests were carried out at the end of the college soccer league season. Conducting psychological measurements at this time allows for an evaluation of the burnout accumulated throughout the season, as well as an understanding of the players' immediate assessments of the coach-athlete relationship and their training experiences. This study was approved by the Ethics Committee. Each participant provided his/her informed consent. The researchers informed each participant that his/her responses would be completely anonymous and confidential.

### Data analysis

Pearson product-moment correlation analysis was used to test the relationship between coach-athlete relationship, training satisfaction, and burnout in collegiate soccer players. Regression analysis was used to test the linear relationship. Considering the fact that Structural Equation Modeling (SEM) can observe the relationship between observed variables and latent variables, as well as simultaneously consider and process multiple dependent variables (Cheng, 2006), it was performed to test the mediating effect of training satisfaction on the relationship between coach-athlete relationship and athlete burnout. Correlation analysis and regression analysis of the data were performed using SPSS 26.0, and SEM analysis was performed using AMOS 24.0. All statistical tests were conducted using a two-tailed test. Set bias-corrected confidence Interval (CI) at 95% and the number of bootstrap samples at 5,000. If CI included zero, the mediation effect was not significant. Conversely, the mediation effect in this study is statistically significant at α = 0.05.

## Results

### Descriptive statistics and correlation

The means and standard deviations of scores in each sub-scale are presented in Table 2.

**Table 2:** Descriptive statistics and correlation coefficients between variables.

**Sub-scales**	**Mean (SD)**	**1**	**2**	**3**	**4**	**5**	**6**	**7**
• Emotion	5.22 (0.79)	1						
• Behavior	5.08 (0.89)	0.35^***^	1					
• Cognition	5.20 (0.76)	0.74^***^	0.35^***^	1				
• TS	3.48 (0.82)	0.37^***^	0.38^***^	0.35^***^	1			
• EPE	2.65 (0.86)	−0.46^***^	−0.44^***^	−0.44^***^	−0.51^***^	1		
• RAA	2.59 (0.85)	−0.46^***^	−0.53^***^	−0.43^***^	−0.55^***^	0.61^***^	1	
• SDV	2.65 (0.85)	−0.47^***^	−0.43^***^	−0.49^***^	−0.45^***^	0.57^***^	0.58^***^	1

^***^p < 0.001.

SD, standard deviation; TS, training satisfaction. EPE, emotional/physical exhaustion. RAA, reduced athletic accomplishment. SDV, sport devaluation.

The mean score of each scale in the coach-athlete relationship scale was above 5, which showed the relationship between coaches and athletes was medium agreeable. The mean score on training satisfaction scale was 3.5, which showed the players had relatively high satisfaction. The mean score of each scale in the athlete burnout questionnaire was about 2.5, which showed that burnout was relatively low. The absolute value of correlation coefficients across each sub-scale were 0.35–0.74, which showed a medium to strong correlation relationship. The results showed that coach-athlete relationship, training satisfaction, and athlete burnout were positively correlated. Therefore, the significant correlation between variables provides a solid basis for subsequent mediation effect testing exploration.

### The mediating role of training satisfaction between coach-athlete relationship and athlete burnout

**Figure 1:**
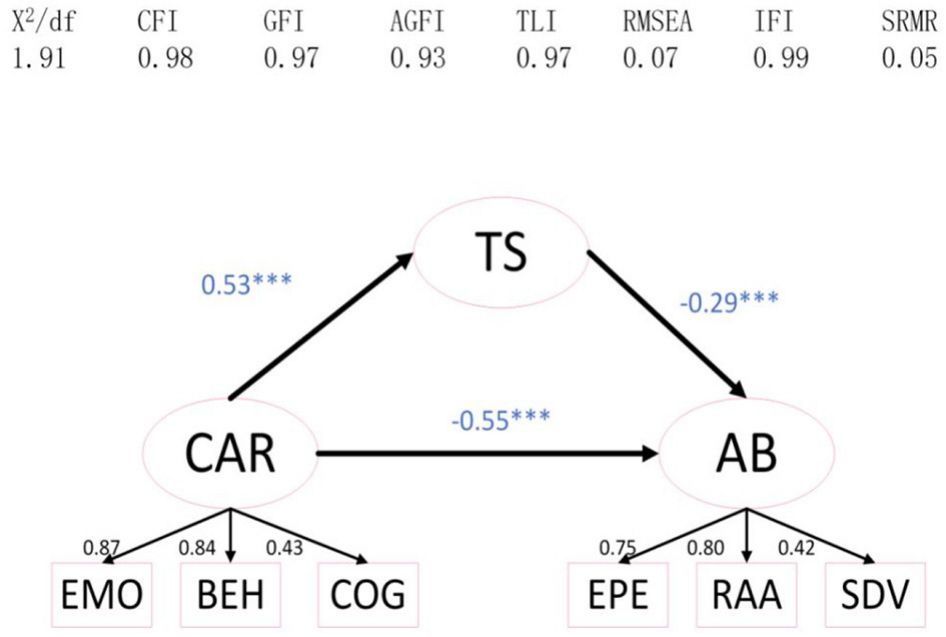
The mediating role of TS in the relationship between CAR and AB. CAR, Coach-Athlete Relationship; AB, athlete burnout; TS, training satisfaction; EMO, emotion; BEH, behavior; COG, cognition; EPE, emotional/physical exhaustion; RAA, reduced athletic accomplishment; SDV, sport devaluation. ***p < 0.001.

To investigate the relationship between coach-athlete relationship, training satisfaction, and athlete burnout, structural equation model analysis was used to analyze the mediating effect, with coach-athlete relationship as the independent variable, athlete burnout as the dependent variable, and training satisfaction as the mediating variable. For instance, after modifying the model, the model fit indices are as follows: X^2^/df = 1.91, GFI = 0.98, CFI = 0.98, TLI = 0.97, AGFI = 0.93, and IFI = 0.99, Additionally, RMSEA = 0.07 and SRMR = 0.05, indicating that the model is acceptable (Figure 1). Coach-athlete relationship has a significantly negative predictive effect on athlete burnout (β = −0.39, *P* < 0.001); meanwhile, training satisfaction has a significantly negative predictive effect on athlete burnout (β = −0.29, *P* < 0.001).

To verify the mediating effect of training satisfaction on coach-athlete relationship and athlete burnout, this study adopted a non-parametric percentile Bootstrap process in SEM analysis to test the mediating effect. The result showed that coach-athlete relationship significantly affected athlete burnout through the mediating effect of training satisfaction [β = −0.15, *P* < 0.001, 95% CI = (−0.228, −0.094)]; effect size was 0.28. The confidence interval not including zero indicated that the mediating effects in this study were statistically significant. All detailed data are shown in Table 3 and the conceptual mediating model is shown in Figure 1.

**Table 3:** Regression coefficients of CAR on AB and TS.

**Effect**	**Path relationship**	**Effect size**	**SE**	***Z***	**Bootstrap 95%CI**
Indirect effect	CAR->TS->AB	0.28	0.03	4.53	(−0.228, −0.094)
Direct effect	CAR->AB	0.72	0.07	5.47	(−0.547, –**-**0.262)
Total effect			0.08	6.94	(−0.709, −0.404)

CAR, coach–athlete relationship; AB, athlete burnout; TS, training satisfaction; SE, standard error; *Z*, Sobel *z*; CI, confidence interval.

## Discussion

### The association among coach-athlete relationship, training satisfaction, and athlete burnout

This study explored the mechanism of the association between coach-athlete relationship, training satisfaction, and athlete burnout. The results showed there are two ways in which the coach-athlete relationship affected athlete burnout. (1) Direct path: coach-athlete relationship directly predicted athlete burnout; (2) Mediating path: coach-athlete relationship indirectly influence predicted athlete burnout through the mediating effect of training satisfaction. The findings of this study suggested that athletes with a high-quality coach-athlete relationship experience greater training satisfaction, which in turn helps reduce the risk of athlete burnout. The findings consistent with the points of Shang and Yang (2021), who found that mental toughness can alleviate stress and bolster the protective impact of interpersonal support on mental health. In this study, training satisfaction similarly mitigated the negative impact of stress and enhanced the inhibitory effect of the support from coaches on burnout (Smith, 1986; Silva, 1990; Moen and Federici, 2017).

### Relationship between coach-athlete relationship and athlete burnout

This study confirms that, overall, there is a harmonious relationship between coaches and collegiate soccer players. Additionally, the study reveals that coach-athlete relationship was negatively linked with athlete burnout. This finding was in line with previous research findings (Cresswell and Eklund, 2007; DeFreese and Smith, 2014; Isoard-Gautheur et al., 2015). In daily training, the supportive and motivating atmosphere established between coaches and athletes can reduce the occurrence of athlete burnout.

From a social science perspective, the social environment factors play a crucial role in the development of athlete burnout (Gould et al., 1996; Moen and Federici, 2017; Lin et al., 2021). The coach-athlete relationship, as a unique social environment, can create a comfortable social interactive environment in which individuals are less likely to experience athlete burnout in sports because mental needs are continuously satisfied. On the other hand, Adie and Jowett (2010) found that when athletes establish a harmonious and supportive relationship with their coaches, athletes are more likely to earn respect from their coaches, thus enhancing their sense of relatedness needs. When coaches provide increased support to athletes, athletes perceive a greater sense of autonomy and control, thereby satisfying their autonomy needs. Additionally, following the training stress syndrome model, the support form coaches can mitigate the negative adaptation of athletes caused by training pressure (Gabana et al., 2017), thereby reducing the levels of athlete burnout. It is evident that coach-athlete relationship could be a factor that can manage the athletes' stress during training.

There are three dimensions in coach-athlete relationship in this study (emotion, cognition, and behavior), and all three dimensions significantly influenced athlete burnout (emotional/physical exhaustion, reduced athletic accomplishment, and sport devaluation). This can be explained by referencing prior research findings (Cresswell and Eklund, 2007; Isoard-Gautheur et al., 2015, 2016). When collegiate athletes feel close to their coaches (i.e., emotion: closeness), their psychological needs are more likely to be fulfilled, thus reducing emotional exhaustion. If collegiate athletes perceive themselves as dependent on their coaches and are willing to maintain a long-term relationship with their coaches (i.e., cognition: commitment), they are more likely to uphold a favorable evaluation of their coach-athlete relationship. This positive perception can serve as a continuous source of motivation, ultimately resulting in a reduction of sport devaluation. When collegiate athletes sense a harmonious partnership with their coaches (i.e., behavior: complementary), it encourages them to exert additional effort for the team, ultimately leading to a decrease in the levels of reduced athletic accomplishment. Furthermore, the findings are similar to those of Guo et al.'s (2021) study. In this study, the direct impact of coach-athlete relationship on athlete burnout was greater, and there might be two reasons causing this situation, (1) The sample sources vary: Their study centered on athletes from sports schools, and their primary social identity is professional athletes, whereas this study's participants were university students, primarily social identity categorized as students. These distinct groups hold varying perspectives on sports (Hellström, 2009), causing collegiate athletes to perceive the coach's commitment differently compared to their professional counterparts. (2) Their research covered various sports, while this study specifically targets soccer. Athletes from different sports disciplines interpret relationships differently (Chen et al., 2009).

### Effect between training satisfaction and athlete burnout

The finding of this study also indicated that training satisfaction was negatively linked with athlete burnout. The findings of this study are similar to those of previous research (Zhan et al., 2015; Zhang, 2015). Training satisfaction is an emotional reaction expressed by athletes after comprehensively evaluating their training and other factors related to training, including aspects such as sports organization and coaching. Establishing high-quality relationships with coaches increases the likelihood of individuals identifying their teams as an integral part, consequently boosting training satisfaction (Jowett and Nezlek, 2012). Moreover, scholars (Moen and Federici, 2017) found that high quality coach-athlete relationship lead to improved training satisfaction and performance outcomes. As the function of training satisfaction for athletes, it is of crucial significance for athletes' sports performance and psychological wellbeing (Xie, 2008), helping reduce the risk of athlete burnout. Thus, it is of great importance for enhancing sport performance and reducing burnout to improve the satisfaction of daily training in collegiate athletes. Certainly, based on the training stress syndrome and cognitive-affective model, to reduce athlete burnout in collegiate soccer players, establish a balanced training regimen that includes both intensity and relaxation is crucial. Furthermore, colleges and universities should endeavor to offer multifaceted support for players' training, aiming to improve their training satisfaction and prevent the occurrence of burnout.

### The mediating role of training satisfaction

This study put forward three new points on the mechanisms of athlete burnout among college soccer players, which enriched the training stress syndrome and cognitive-affective model by providing new explanations for alleviating athlete burnout from the perspective of interpersonal relationships and training perceptions. Firstly, this study introduced a perspective that has not been previously explored by previous researchers on athlete burnout by reporting the positive associations between coach-athlete relationship and training satisfaction. Based on the finding that training satisfaction is a mediator, this study provides new support that the coach-athlete relationship can improve training satisfaction (Jowett and Nezlek, 2012; Davis et al., 2019; Cho and Baek, 2020). Collegiate athletes often train in high-pressure environments. In this situation, the care and support provided by their coaches are crucial factors for reducing athletes' stress levels and serve as essential predictors of training satisfaction (Paul and Tim, 2010; Gabana et al., 2017; Zhang et al., 2021). When the relationship between collegiate athletes and their coaches is at a high level, collegiate athletes are more likely to engage in discussions with their coaches and seek assistance when they are in stress. Timely care and support from coaches can enhance collegiate athletes' self-regulation and, in turn, increase collegiate athletes' training satisfaction. Secondly, the study also found that the mediating role of training satisfaction in the relationship between emotion and behavior in coach-athlete relationship and training athlete burnout. In summary, according to training stress syndrome and cognitive-affective model, when athletes experience burnout due to negative adaptation to training pressures, positive relationships between coaches and athletes can enhance athletes' psychological resilience (Gabana et al., 2017; Sindermann et al., 2020), such as enhance athletes' sense of self-efficacy contributing to improving athletes' psychological coping abilities, thereby reducing the impact of athlete burnout (Zhang et al., 2021). Furthermore, a balanced training regimen can mitigate the negative impacts of training fundamentally. Additionally, positive relationships between coaches and athletes facilitate effective communication, enabling athletes to have a clearer understanding of training objectives, enhancing their subjective training experiences, and ultimately reducing athlete burnout. Most previous studies have examined how athletes' motivation on athletes' motivation affect athlete burnout (Ishak et al., 2013; Madigan et al., 2016; Graa et al., 2021), this study examined the influence of coach-athlete relationship and training satisfaction on the perception of athlete burnout, taking into account both external and internal factors. The findings demonstrate that both the interpersonal relationships of athletes and their subjective training experiences can affect the extent to which they perceive burnout. Finally, this study delved into a less-explored demographic, focusing on collegiate athletes. Its objective is to uncover the factors influencing athlete burnout, particularly examining the impact of the coach-athlete relationship and training satisfaction. Ultimately, this research seeks to offer a mediating model for the effective alleviation of athlete burnout among collegiate athletes, a subject that has received limited consideration in prior studies.

### Practical implication

The findings of the study have essential practical advantages for intervening in athlete burnout from collegiate athletes and coaches. First of all, coaches should ensure timely communication with collegiate athletes during training. Extended periods of high-intensity training or the lead-up to competitions can place collegiate athletes under considerable stress. Adequate social support and effective communication can help mitigate this pressure (Raedeke and Smith, 2001), thus reducing the likelihood of athlete burnout. Next, there should be a strong emphasis on nurturing a positive team culture. The development of team culture contributes to strengthening team cohesion (Xie and Wu, 2013), and team cohesion is a crucial factor in enhancing athlete satisfaction (Ma and Wang, 2006). Hence, the importance of fostering team culture cannot be overlooked. Teams should foster a culture of self-management and self-reflection, encouraging the transition from external management to self-management. This internalization of ongoing training as a self-regulated behavior helps boost collegiate athletes' enthusiasm for participation in training. Besides, regular team-building activities for representatives should be organized to help collegiate athletes clarify their goals, instill the right life values, and strengthen their sense of belonging. This fosters mutual respect among collegiate athletes and between collegiate athletes and coaches within a harmonious coach-athlete relationship. Moreover, players should incorporate mental training techniques into their routines to enhance their psychological resilience (Mitchell et al., 2024). Additionally, when players realize they are struggling, they should seek support from family, friends, teammates, and coaches immediately to boost their self-confidence. Players should manage their time wisely, striking a balance between life, academics, and training, setting both short-term and long-term training goals, and maintaining a healthy perspective on wins and losses (Marks et al., 2022). Not only that, collegiate athletes should view setbacks as opportunities for improvement and believe in their potential for success. Based on the findings of this study and by training stress syndrome and cognitive-affective model, it is reasonable to suggest that the aforementioned strategies for mitigating athlete burnout are highly practicable.

## Conclusion

Coach-athlete relationship has a significantly negative predictive effect on athlete burnout, and training satisfaction also has a significantly negative predictive effect on athlete burnout; coach-athlete relationship has a significantly positive predictive effect on training satisfaction; meanwhile, training satisfaction plays a mediating role in the relationship between emotion and behavior in coach-athlete relationship and athlete burnout, which means training satisfaction can enhance the weakening effect of coach-athlete relationship on athlete burnout. This study, from the perspective of collegiate athletes and based on training stress syndrome, elucidates the internal mechanisms of how the coach-athlete relationship and training satisfaction influence athlete burnout, providing theoretical guidance for alleviating athlete burnout.

Future research could adopt a bidirectional perspective from both coaches and athletes to more comprehensively observe the quality of the coach-athlete relationship. Furthermore, from the perspective of the causes of athlete burnout, future research could explore factors such as social support and family support, which can mitigate negative training adaptation among athletes. This exploration would uncover more methods to alleviate athlete burnout and enhance athletes' performance throughout their careers.

## Limitations and further directions

Firstly, regarding the research design, this study is a cross-sectional study, making it difficult to determine the specific causal relationships between variables. Future research could consider longitudinal studies to more clearly investigate the causal relationships between variables over time. Secondly, about variable selection in the study, the factors influencing athlete burnout involve multiple aspects. This study only focuses on the coach-athlete relationship and training satisfaction. Future research could further explore other aspects that may affect athlete burnout, such as social support. Finally, the cronbach's α of cognition component in the coach-athlete relationship scale was lower in this study compared to a previous study (Guo et al., 2021). This discrepancy could be attributed to differences in the sample structure. While Guo's study included athletes from multiple sports, our study focused solely on college soccer players. The limitation suggests the necessity to expand the sample structure in future studies to enhance the applicability of the research findings.

## Data availability statement

The raw data supporting the conclusions of this article will be made available by the authors, without undue reservation.

## Ethics statement

The studies involving humans were approved by the ethics committee of Sichuan university (approval no.KS20240023). The studies were conducted in accordance with the local legislation and institutional requirements. The participants provided their written informed consent to participate in this study.

## Author contributions

LJia: Conceptualization, Data curation, Investigation, Software, Visualization, Writing – original draft, Writing – review & editing. LJin: Funding acquisition, Methodology, Supervision, Writing – review & editing.
